# Multi-scale information bottleneck with confidence-weighted decision fusion for robust breast ultrasound lesion classification

**DOI:** 10.3389/fonc.2026.1857871

**Published:** 2026-07-08

**Authors:** Gang Liu, Sijia Chen, Yaling Zhu, Hui Zhang, Yan Li, Qingjie Dong

**Affiliations:** 1 Department of Medical Ultrasound, Nanchong Central Hospital (Beijing Anzhen Hospital, Nanchong Hospital), Nanchong, Dazhou, China; 2 Dazhou Central Hospital, Department of Ultrasound Medicine, Sichuan, Dazhou, China; 3 Dazhou Central Hospital, Information Department, Sichuan, Dazhou, China

**Keywords:** breast ultrasound lesion, confidence-weighted fusion, deep learning, information bottleneck, multi-scale

## Abstract

Breast cancer remains a leading cause of cancer-related mortality among women, and breast ultrasound (BUS) offers a widely used, non-invasive modality for early detection and treatment planning. However, BUS-based computer-aided diagnosis is often hindered by speckle noise, device-dependent intensity variations, and substantial heterogeneity in lesion size, contrast, and appearance, which can degrade the robustness of conventional CNN classifiers. In this study, we propose a multi-scale information-bottleneck-guided classification framework tailored to BUS lesion analysis. A ResNet backbone with a feature pyramid network (FPN) extracts hierarchical multi-scale representations that jointly encode fine lesion details and global structural context. An information bottleneck (IB) module is attached to each FPN level to learn channel-wise gating masks from global statistics, suppressing background glandular textures and scanner-related artifacts while preserving discriminative lesion cues; auxiliary classifiers provide deep supervision at each scale, producing per-level class-posterior estimates. These scale-specific predictions are then aggregated via confidence-weighted decision-level fusion to obtain a unified, uncertainty-aware output. Experiments on breast ultrasound datasets demonstrate improved classification performance and enhanced robustness over representative CNN baselines, with notable benefits in challenging small and low-contrast lesion scenarios, supporting the framework’s potential for clinically applicable BUS-based screening.

## 1 Introduction

Breast cancer is one of the most prevalent malignancies among women worldwide and remains a leading cause of cancer-related mortality, despite notable advances in screening and systemic therapies Waks and Winer (1) Xiong et al. (2) Lewis et al. (3). In routine clinical practice, breast ultrasound (BUS) Al-Dhabyani et al. (4) Tanaka et al. (5) is widely used as a complementary modality to mammography, particularly for women with dense breasts, pregnant or lactating patients, and those requiring frequent follow-up. Compared to mammography and MRI, BUS is radiation-free, relatively low-cost, and easily accessible in primary and secondary care institutions, making it become a cornerstone of early detection and triage in many healthcare systems. However, BUS interpretation (6) is highly operator-dependent and subject to considerable inter- and intra-observer variability, especially when lesions are small, low-contrast, or embedded in heterogeneous glandular tissue. This variability can delay diagnosis, lead to unnecessary biopsies, and complicate treatment planning, underscoring the clinical need for robust, reproducible computer-aided diagnosis (CAD) systems to support radiologists in everyday decision-making.

Over the past decade, a variety of BUS-based CAD methods have been proposed, ranging from traditional machine learning Huang et al. (7); Mishra et al. (8); kaba Gurmessa and Jimma (9) models built on handcrafted texture, shape, and intensity descriptors to deep learning Kiran et al. (10), Dan et al. (11), Li et al. (12) approaches that automatically learn discriminative representations from raw images. Classical methods depend heavily on manual feature engineering and often fail to generalize across different scanners and acquisition protocols. More recent CNN-based Tagnamas et al. (13), Çakmak and Pacal (14) classifiers have demonstrated clear performance gains, yet most of them implicitly treat BUS images as natural images and mainly rely on single-scale or shallow multi-scale feature hierarchies. In the presence of severe speckle noise, device-dependent intensity shifts, and large variability in lesion size and appearance, such architectures tend to overfit background glandular textures or scanner-specific artifacts rather than focusing on lesion-centered cues. Furthermore, existing multi-scale strategies generally fuse features at an intermediate representation level or use simple score averaging across scales, without explicitly modeling the reliability of each scale’s prediction or the uncertainty arising from noisy, heterogeneous inputs. These limitations contribute to unstable performance across datasets, imaging devices, and clinical environments, which hinders the deployment of CAD systems in real-world breast clinics.

To address these challenges from a perspective aligned with clinical BUS workflows, we propose a multi-scale information-bottleneck-guided classification framework tailored for breast lesion analysis. Building on a ResNet He et al. (15) backbone and a feature pyramid network (FPN), our method constructs multi-scale feature maps that jointly capture fine-grained lesion details and global structural context, while an information bottleneck (IB) module at each pyramid level learns channel-wise gating masks to suppress residual speckle noise, homogeneous glandular patterns, and scanner-specific artifacts. Each IB module is equipped with an auxiliary classifier, providing deep multi-scale supervision that encourages the bottlenecked features to retain only the most lesion-relevant information. At the decision level, we further interpret the per-scale outputs as class-posterior estimates and fuse them via a confidence-weighted aggregation scheme, yielding a unified, uncertainty-aware prediction that better reflects the consensus across complementary scales. In this way, the proposed framework not only enhances the robustness and interpretability of BUS-based classification, but also aligns with the clinical demand for reliable, device-agnostic tools to support precision breast cancer screening and diagnosis.

The main contributions are summarized as follows:

1. We design a BUS-specific classification architecture that couples a ResNet backbone with a feature pyramid network, enabling joint modeling of fine-grained lesion details and global structural context. By explicitly targeting common BUS artifacts—speckle noise, heterogeneous glandular tissue, and device-dependent intensity shifts—the framework is tailored to the practical challenges of breast cancer diagnosis in routine ultrasound workflows.
2. We introduce information bottleneck (IB) modules at each pyramid level to learn channel-wise gating masks that suppress background glandular patterns and scanner-specific artifacts while preserving lesion-centered, class-discriminative cues. Each IB module is supervised by an auxiliary classifier to enforce multi-scale, lesion-relevant representations, and the resulting per-scale class posteriors are fused via a confidence-weighted aggregation scheme at the decision level, yielding a unified, uncertainty-aware prediction.
3. Extensive experiments on breast ultrasound datasets show that the proposed framework consistently outperforms strong CNN-based baselines in terms of accuracy, sensitivity, and AUC, with particularly pronounced gains for small and low-contrast lesions. Additional analyses demonstrate improved robustness under cross-dataset evaluation, highlighting the potential of the proposed method as a reliable CAD tool for precision breast cancer screening and diagnosis.

The rest of paper is organized as follows: In Section 2, we review the related literature. In Section 3, we describe our proposed methods in detail. In section 4, we conduct several experiment to verify the superiority of our proposed method. Finally, we concluded our work in section V.

## 2 Related work

In this section, we review previous studies on breast ultrasound (BUS)-based computer-aided diagnosis from a problem-oriented perspective. Rather than simply enumerating handcrafted-feature methods, machine-learning pipelines, and deep learning models in chronological order, we focus on several unresolved issues that are directly related to the proposed framework: robust lesion representation under ultrasound-specific artifacts, multi-scale modeling of heterogeneous lesion appearances, compact representation learning, and reliable fusion of scale-specific diagnostic evidence.

Early BUS-based CAD systems predominantly relied on handcrafted descriptors combined with classical classifiers. Typical feature sets included first- and second-order intensity statistics, gray-level co-occurrence matrix (GLCM) and other texture measures, contour- and shape-related descriptors, and BI-RADS-derived morphological indices that encode lesion margin, echogenicity, and posterior acoustic features (7, 16, 17). These descriptors were usually fed into support vector machines (SVM), k-nearest neighbors (k-NN), random forests, or other conventional classifiers to distinguish benign from malignant lesions (18). Although such pipelines provided early evidence that quantitative ultrasound features can assist radiologists, their performance strongly depends on manually designed features and is sensitive to acquisition parameters, vendor-specific post-processing, and operator variability. As a result, their discriminative ability often degrades when lesions are small, low-contrast, or embedded in heterogeneous glandular tissue (19, 20).

Radiomics-style and machine-learning-driven pipelines further extended handcrafted BUS analysis by extracting higher-order texture, wavelet, and shape features, followed by feature selection and statistical or machine-learning classifiers (21, 22). These methods can capture richer lesion characteristics than purely handcrafted descriptors, but they still depend on pre-defined feature families and usually separate feature extraction from classifier optimization. This decoupled design makes it difficult to jointly adapt the entire pipeline to BUS-specific artifacts, such as speckle noise, device-dependent intensity shifts, and blurred lesion boundaries. Therefore, although handcrafted and radiomics-based methods are interpretable, they remain limited in modeling complex lesion appearances and heterogeneous imaging conditions.

Deep convolutional neural networks (CNNs) have become the mainstream approach for BUS lesion classification because they can automatically learn hierarchical feature representations from image data Afrin et al. (23). Common natural-image backbones, such as AlexNet, VGG, ResNet, DenseNet, Inception, Xception, and EfficientNet, have been adapted to BUS classification through transfer learning and end-to-end optimization Kiran et al. (10)Arooj et al. (24). Subsequent studies further explored deep feature fusion, multi-task learning, segmentation-assisted classification, and attention mechanisms to enhance lesion localization and suppress surrounding tissue interference (25–27). In particular, Hotiet et al. proposed a dual-module deep learning framework for ultrasound-based breast cancer diagnosis by combining lesion classification and segmentation, further demonstrating the value of jointly considering diagnostic prediction and lesion localization in BUS analysis (28). These deep learning methods have achieved substantial improvements in accuracy, sensitivity, and AUC on curated BUS datasets. However, many of them still rely on single-scale global representations or standard feature hierarchies originally designed for natural images, which may be insufficient for handling the large variations in lesion size, depth, contrast, and appearance commonly observed in BUS examinations.

Recently, transformer-based and hybrid CNN-transformer methods have received increasing attention in BUS image analysis because self-attention is able to capture long-range dependencies and global contextual relationships. Gheflati and Rivaz introduced Vision Transformer models for breast ultrasound image classification and showed that ViT-based models can achieve competitive performance compared with CNN-based approaches Gheflati and Rivaz (29). Hybrid-MT-ESTAN further combined CNNs with Swin Transformer components for BUS tumor classification and segmentation, demonstrating the benefit of integrating local convolutional patterns with global contextual modeling? HCTNet adopted a hybrid CNN-transformer design for breast ultrasound lesion segmentation, where transformer blocks were used to capture long-range dependencies while CNN components preserved local details (30). More recently, SW-ForkNet introduced a Swin Transformer-based fork architecture for breast tumor classification by combining spatial, semantic, and long-context features (31). These studies indicate that transformer-based global modeling and CNN-transformer hybrid structures are promising for BUS analysis. Nevertheless, such models often require relatively large datasets and computational resources, and self-attention itself does not explicitly suppress ultrasound-specific nuisance information, such as speckle noise, background glandular textures, and scanner-related intensity variations.

Recent multi-scale and attention-based BUS methods attempt to alleviate these problems by enhancing lesion-related regions and integrating features from different resolutions (32, 33). Such designs are useful because small lesions usually require high-resolution local details, whereas large or low-contrast lesions may depend more on broader structural context. Nevertheless, existing multi-scale strategies often perform feature fusion through concatenation, summation, or simple score averaging. These operations can aggregate complementary information but usually do not explicitly evaluate whether each scale-specific feature or prediction is reliable under noisy and heterogeneous BUS imaging conditions. In addition, attention mechanisms can down-weight irrelevant regions or channels, but they usually operate as empirical weighting modules without an explicit information-theoretic constraint to suppress speckle noise, background glandular textures, and scanner-dependent artifacts (34, 35).

The information bottleneck (IB) principle provides a useful perspective for addressing this limitation. Its core idea is to learn compact representations by reducing input-dependent redundant information while preserving task-relevant discriminative information Hu et al. (36). This is particularly suitable for BUS images, where lesion cues are frequently entangled with ultrasound-specific nuisance factors, including speckle noise, acoustic shadows, homogeneous tissue responses, and device-dependent intensity variations. However, IB-based representation learning has not been sufficiently contextualized in existing BUS classification studies. Most existing BUS-CAD methods rely on attention, saliency, or feature aggregation to enhance lesion regions, but few explicitly introduce an IB-guided constraint into a multi-scale feature pyramid. Therefore, there remains a need to learn scale-specific compact representations that retain lesion-centered information while suppressing redundant and noise-dominated channels.

Another unresolved issue is how to fuse heterogeneous predictions from different feature scales. Existing ensemble or multi-scale BUS methods usually aggregate features or scores using fixed weights, concatenation, summation, or simple averaging (32, 33). Although these strategies can improve performance, they implicitly assume that all scales or branches are equally reliable. In clinical BUS images, this assumption may not hold. For example, high-resolution features may be more reliable for small lesions, whereas deeper semantic features may be more useful for large or structurally complex lesions. When predictions from different scales have unequal confidence, simple averaging may introduce noise from unreliable branches. In medical image analysis, uncertainty quantification has been increasingly studied to improve the reliability, interpretability, and clinical acceptability of deep learning models (37, 38). These studies motivate a decision-level fusion strategy that can adaptively weight scale-specific posterior predictions according to their reliability.

In summary, existing BUS-based CAD methods have progressed from handcrafted descriptors and machine-learning classifiers to CNN-based, transformer-based, multi-scale, and attention-enhanced deep models. However, three limitations remain insufficiently addressed. First, many methods improve feature extraction but do not explicitly suppress ultrasound-specific nuisance information under an information-theoretic principle. Second, existing multi-scale, attention-based, and transformer-based approaches often lack scale-specific compact representation learning tailored to BUS lesion characteristics. Third, conventional fusion strategies usually ignore the reliability and uncertainty differences among scale-specific predictions. To address these gaps, we propose a multi-scale information-bottleneck-guided classification framework with confidence-weighted decision-level fusion. The ResNet–FPN backbone extracts complementary multi-scale features, the scale-specific IB modules enforce compact lesion-centered representations, and the confidence-weighted fusion module adaptively aggregates per-scale posterior probabilities according to their reliability. This design directly targets the unresolved tension among multi-scale lesion representation, nuisance-information suppression, and uncertainty-aware decision fusion in robust BUS lesion classification.

## 3 The proposed method

In this section, we describe our proposed method in detail, including the overall framework, the design rationale behind each network component, and the information-bottleneck mechanisms that enable robust and lesion-focused breast ultrasound cancer classification.

### 3.1 The overview of the proposed methods

**Figure 1:**
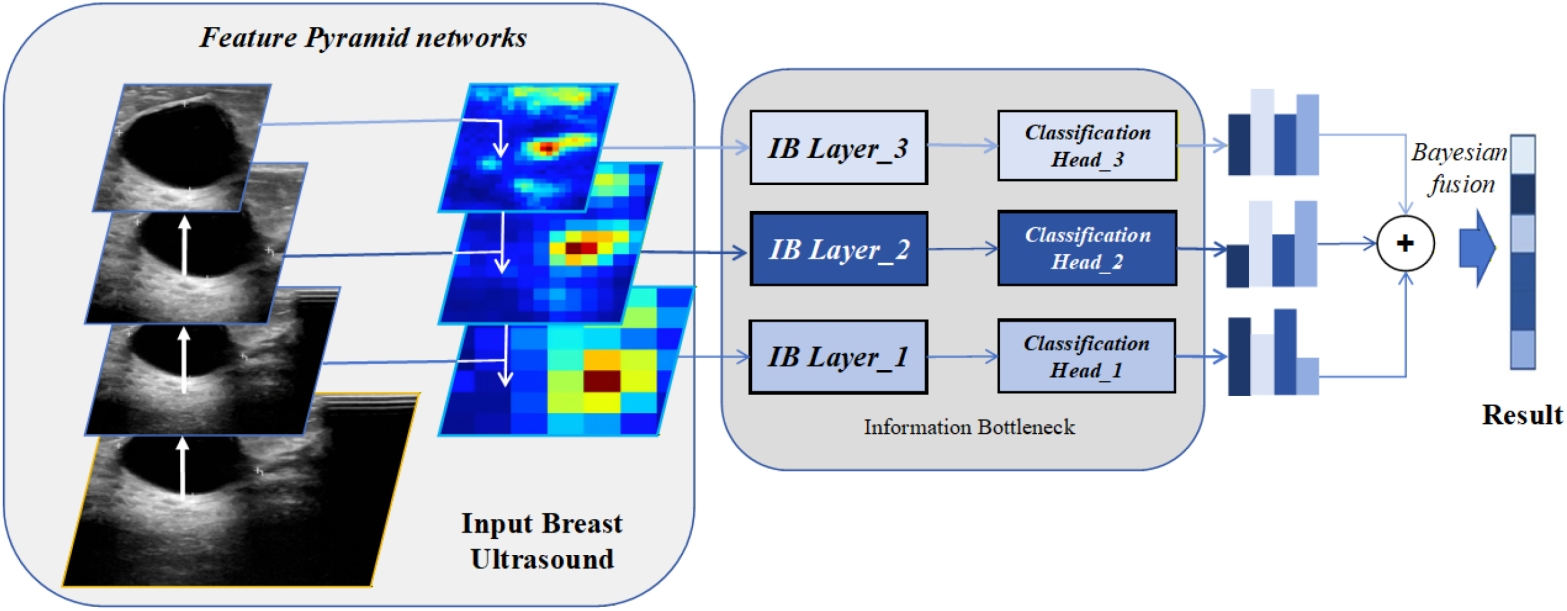
Overview of the proposed multi-scale information-bottleneck-guided classification framework. A ResNet backbone extracts hierarchical features from the input BUS image, and an FPN constructs three pyramid levels (P3, P4, P5) to encode multi-scale lesion characteristics. Each level contains an information bottleneck (IB) module that performs channel-wise gating to suppress noise, scanner-specific artifacts, and background glandular textures. Auxiliary classifiers provide deep supervision at each scale, and the resulting per-scale posterior probabilities are fused via a confidence-weighted aggregation scheme to obtain a unified, uncertainty-aware prediction.

The proposed framework is a multi-scale classifier tailored for breast ultrasound (BUS) images, as illustrated in **Figure 1**. Given an input BUS image X, a convolutional backbone first extracts a hierarchy of feature maps at different spatial resolutions. These multi-level features encode complementary information: shallow layers preserve fine structural details of small masses, whereas deeper layers capture high-level semantic context around larger lesions.

On top of these backbone outputs, a feature pyramid structure aggregates information across scales to form three classification levels (from high to low resolution). Each pyramid level produces a feature map that is fed into a lightweight prediction block followed by an Information Bottleneck (IB) module. The prediction block standardizes the channel dimension and prepares task-oriented features, while the IB module performs channel-wise filtering to suppress noise-dominated responses and retain only lesion-relevant channels. This design is repeated in a parallel manner for all pyramid levels, ensuring that every scale benefits from the same IB-driven compression mechanism.

Finally, the IB-refined features at each level are passed to the corresponding classifier which output class probabilities for lesion categories (e.g., benign vs. malignant) for that scale. Predictions from all levels are then fused at the decision level using a confidence-weighted method, which dynamically weighs each scale’s prediction based on its reliability and confidence. The final prediction is derived by aggregating the class posteriors from each scale, with more reliable scales contributing more to the final result.

### 3.2 Data preprocessing

To improve the visual quality of breast ultrasound (BUS) images and stabilize the input distribution, we perform two preprocessing operations: speckle denoising and intensity normalization.

Speckle denoising: Since BUS images contain multiplicative speckle noise, a speckle-reducing median filter is applied to each input image *X*. For a pixel position (*i,j*), the denoised output *X*(*i,j*) is computed as in Equation 1:

(1) $$\hat{X}(i,j)=\text{median }\{X(m,n)|(m,n)\in {\text{Ω}}_{i,j}\}$$

where ${Ω}_{i,j}$ denotes a local neighborhood window. In our setting, we use the window size of 3 × 3. The 3×3 median filter was adopted because it provides a simple and edge-preserving preprocessing strategy for suppressing local speckle noise while avoiding excessive smoothing of lesion boundaries. This is important for BUS lesion classification, since boundary irregularity and subtle low-echo regions are diagnostically relevant. Compared with more complex denoising methods such as non-local means or anisotropic diffusion filtering, the median filter introduces fewer tunable parameters, has lower computational overhead, and can be consistently applied to all compared models without adding additional optimization complexity. Therefore, it was selected as a lightweight preprocessing step rather than as a separate enhancement module.

Normalization: After denoising, pixel intensities are scaled to a fixed dynamic range [0,1],This reduces device-dependent gray-level variations and ensures stable gradient magnitudes during training. To further reduce inter-image intensity shifts, each image is standardized to zero mean and unit variance, it is defined as in Equations 2, 3:

(2) $$X_{\text{norm}}=\frac{\hat{X}-\text{min}\;(\hat{X})}{\text{max}\;(\hat{X})-\text{min}\;(\hat{X})}$$

(3) $$X_{\text{std}}=\frac{X_{\text{norm}}-\theta }{\sigma }$$

where *θ* and *σ* denote the mean and standard deviation of *X*_norm_.

### 3.3 The network structure

To address the inherent challenges of breast ultrasound (BUS) imaging – including strong speckle noise, low contrast, and large variation in lesion size – we adopt a ResNet backbone combined with a Feature Pyramid Network (FPN) and Information Bottleneck (IB) modules. The goal is to learn multi-scale, lesion-centric representations while suppressing noise-dominated and device-dependent features.

Given a preprocessed BUS image *X* ∈ *R^H^*^×^*^W^*, the ResNet backbone extracts a hierarchy of feature maps {*C*_2_*,C*_3_*,C*_4_*,C*_5_},with progressively lower spatial resolution and higher semantic abstraction. Each stage is composed of multiple residual blocks. A generic residual block takes an input feature map *x* and outputs, it is defined as in Equation 4:

(4) y=F(x;W)+x

where *F*(·) is a residual function implemented by a sequence of convolution–batch normalization–ReLU layers with learnable parameters *W*. Residual learning is particularly beneficial in BUS images for two reasons: 1) Robust optimization under noise: strong speckle and low contrast make optimization unstable. The identity skip connection in (4) stabilizes gradient propagation and prevents the network from overfitting to noisy patterns; 2) Multi-level lesion description: shallow features preserve fine structural details crucial for tiny, low-echo nodules, whereas deeper features encode high-level context (e.g., architectural distortion, surrounding tissue) needed to recognize irregular malignant masses. Thus, the ResNet backbone provides a natural hierarchical representation matching the multi-scale, heterogeneous nature of breast lesions.

Breast lesions in ultrasound vary from a few millimeters to several centimeters and may appear at different depths within dense glandular tissue. Moreover, their boundaries are often blurred by speckle noise and low contrast. To enhance lesion-related features under such conditions and to handle the large scale variation, we employ a top–down Feature Pyramid Network (FPN) built on {*C*_2_*, C*_3_*, C*_4_*, C*_5_}, from the ResNet backbone.

First, lateral 1×1 convolutions produce intermediate features as in Equation 5.

(5) $$L_{l}={\text{Conv}}_{1\times 1}(C_{l}),\;l\in \{2,3,4,5\}$$

Then a top–down pathway with upsampling and fusion is used to generate the pyramid features {*P*_2_*, P*_3_*, P*_4_*, P*_5_} as defined in Equations 6, 7:

(6) $$P_{5}={\text{Conv}}_{3\times 3}(L_{5})$$

(7) $$P_{l}={\text{Conv}}_{3\times 3}(L_{l}+UpSample(P_{l+1}))$$

where *UpSample*(·) denotes a 2× upsampling operator and ${\text{Conv}}_{3\times 3}$ operator was used to reduce aliasing after fusion.

Through this top–down feature enhancement, each *P_l_*carries high-resolution spatial details and rich semantic context. The high-resolution component is crucial for preserving subtle intensity transitions and fine edges, which allows the network to better delineate small, poorly contrasted nodules that are easily blurred by speckle noise and dense glandular tissue. At the same time, the semantic context inherited from deeper layers helps the model capture global lesion configuration, including overall shape, margin irregularity, and infiltrative growth patterns that are typical of malignant tumors. By jointly encoding these local and global cues at multiple scales, the FPN effectively strengthens lesion saliency under low-contrast, low-SNR conditions. However, the resulting multi-scale features may still encode components that are unrelated to the classification task such as residual noise, background texture, and device-dependent artifacts, which motivates the introduction of information-bottleneck modules to further compress task-irrelevant information and retain only lesion-critical cues in the subsequent stages.

### 3.4 The information bottleneck module

**Figure 2:**
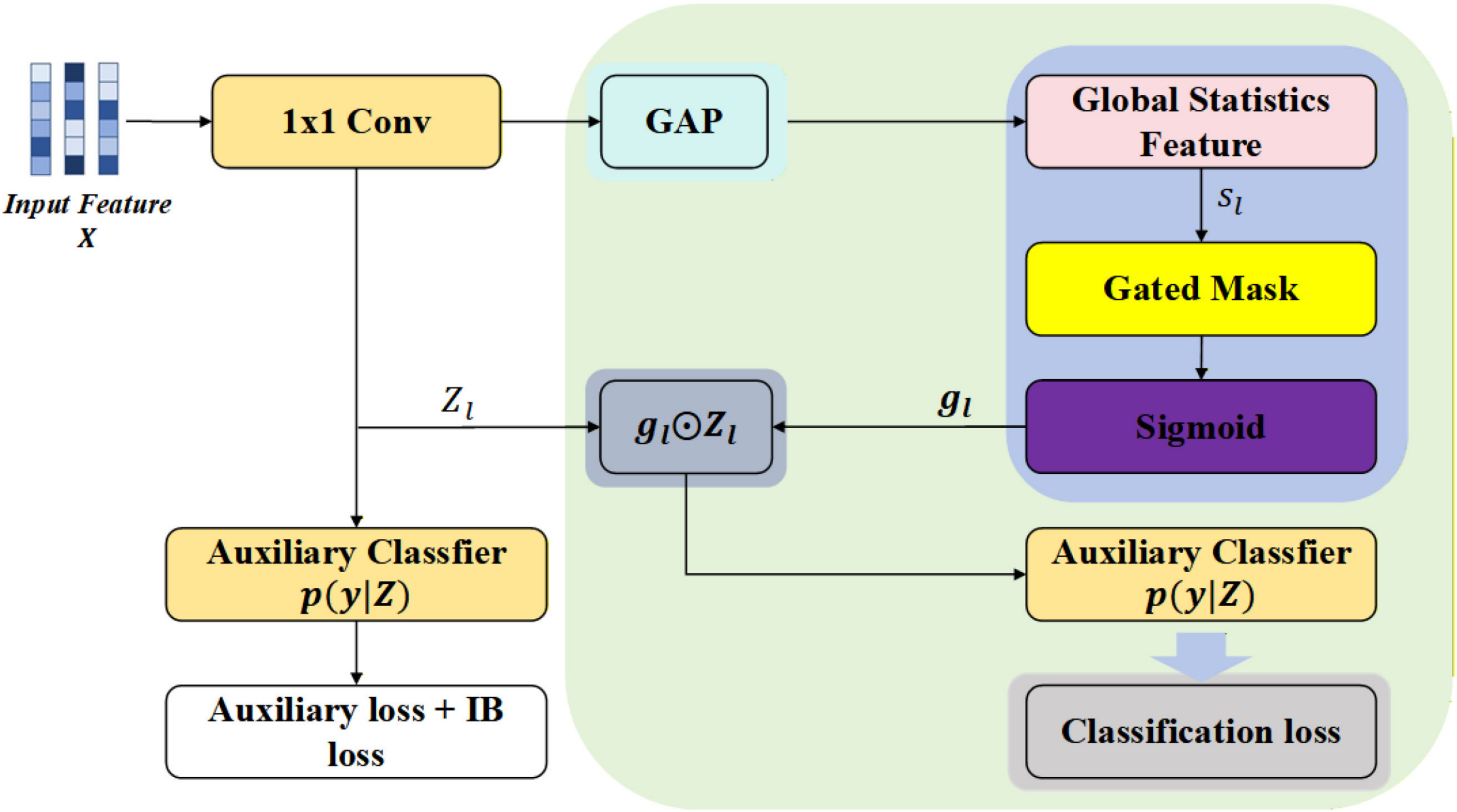
Overview of the information bottleneck (IB) module and its role in the feature suppression process. The input low-dimensional feature map passes through a 1×1 convolution layer, followed by Global Average Pooling (GAP). The output is then processed through a sigmoid activation function and combined with an auxiliary branch to generate the suppression factor. The feature map is then suppressed accordingly, leading to the final feature output, which contains reduced background noise and irrelevant information, with focus on the discriminative lesion-related features.

To further refine the multi-scale features produced by the ResNet–FPN backbone and suppress the components that are unrelated to the breast lesion classification task, we introduce an Information Bottleneck (IB) module at each scale of the feature pyramid. As showed in **Figure 2**, this module aims to selectively extract task-relevant feature representations by suppressing residual speckle noise, background glandular textures, and device-dependent intensity variations that remain after multi-scale aggregation, while retaining the most discriminative lesion-related cues.

Given a pyramid feature map $Pl\;\in \;R^{C\times Hl\times Wl}$ scale *l*, we first introduce a task-aware intermediate representation by applying a 1×1 convolution:

(8) $$Z_{l}={\text{Conv}}_{1\times 1}(P_{l})$$

This projection serves two purposes: 1) reducing feature redundancy by linearly reweighted channels; 2) providing a compact descriptor that can be directly evaluated by an auxiliary classifier during training. In our implementation, $Z_{l}$ is obtained as a deterministic projection through the 1 × 1 convolution, and no stochastic sampling or reparameterization operation is performed; therefore, the KL-based term is used as a regularization surrogate to encourage compact feature representations rather than as an exact stochastic variational inference procedure.

To implement the information bottleneck constraint and emphasize task-relevant information, we derive a channel-wise gating mask from the global statistics of the feature map:

(9) $$s_{l}=\text{GAP }(Z_{l}),\;g_{l}=\sigma (\gamma_{l}⊙s_{l}+b_{l})$$

where *GAP*(·) denotes global average pooling, *σ*(·) is the sigmoid function, and *γ_l_, b_l_*∈ *R^C^* are learnable parameters. The IB-refined feature is obtained as shown in Equation 10:

(10) $${\tilde{Z}}_{l}=Z_{l}⊙g_{l}$$

This gating operation suppresses channels that predominantly encode ultrasound-specific nuisance factors—such as speckle noise patterns, homogeneous tissue responses, or scanner-dependent contrast fluctuations—and retains channels that consistently contribute to lesion classification. To encourage the network to learn a representation that is both compact and lesion-centric, we attach an auxiliary classifier to each intermediate representation *Z_l_*. It is defined as in Equation 11:

(11) $${\hat{y}}_{l}=\text{Softmax }(W_{l}Z_{l}+b_{l})$$

The proposed module is designed from the perspective of feature compression and task-relevant information preservation, following the information bottleneck principle Tishby et al. (39). Structurally, the 1×1 convolution in Equation 8 first projects the FPN feature *P_l_*into a compact scale-specific representation $Z_{l}$, which serves as the bottleneck representation at the *l*-th pyramid level. The global average pooling operation in Equation 9 summarizes the channel-wise global response of $Z_{l}$, and the sigmoid gating function generates a channel-wise suppression mask. This gating form is related to channel-attention mechanisms such as squeeze-and-excitation networks (40), since both use global feature statistics and sigmoid gating to reweight feature channels. However, our module differs in its objective and usage: it is attached to each FPN level and jointly optimized with auxiliary classification supervision and a KL-based variational compression term. Therefore, the module is designed not only for channel recalibration, but also for task-guided feature compression and lesion-discriminative information preservation.

The auxiliary supervision introduces an additional IB-oriented loss term:

(12) $$L_{\text{aux}}=\sum_{l}\text{CE }(y,{\hat{y}}_{l})+I(Z_{l};X)-\beta I(Z_{l};y)$$

where *CE*(·) denotes the cross-entropy loss and *y* is the ground-truth label. It should be noted that the exact mutual information terms $I(Z_{l};X)$ and *I* $\left(Z_{l};y\right)$ are not directly computed in our implementation, since exact mutual information estimation is generally intractable in deep neural networks. Instead, we adopt a practical surrogate implementation following the variational information bottleneck principle. Specifically, the input-related compression term $I(Z_{l};X)$is approximated by a variational upper bound:

(13) $$I(Z_{l};X)\leq E_{p(X)}[D_{\text{KL}}(q_{\phi }(Z_{l}|X)\|p(Z_{l}))].$$

where $q_{\phi }(Z_{l}|X)$ denotes the variational posterior of the scale-specific representation and $p(Z_{l})$ denotes the prior distribution. In our implementation, $p(Z_{l})$ is set as a standard Gaussian prior. The variational posterior $q_{\phi }(Z_{l}|X)$ is parameterized by the scale-specific projection branch that generates the compact representation $Z_{l}$. Therefore, the KL-divergence term acts as a tractable surrogate for compressing redundant input- dependent information. For the label-related discriminative term $I(Z_{l};y)$, we do not directly estimate mutual information. Instead, we optimize it through the auxiliary classification loss $\text{CE }(y,{\hat{y}}_{l})$. Minimizing this loss encourages each scale-specific representation to preserve lesion-relevant discriminative information.

In implementation, Equation 12 is optimized through the KL-based variational compression term and the auxiliary cross-entropy loss. Therefore, Equation 12 is implemented as a tractable variational surrogate rather than by directly estimating exact mutual information.

### 3.5 Confidence-weighted decision-level fusion

At the decision level, we treat the classifiers attached to different pyramid scales as a set of complementary scale-specific predictors that provide heterogeneous but correlated class-posterior predictions. Let *p_j_*denote the posterior probability vector produced by the *j*-th scale-specific classifier. Since different FPN levels may have different reliability for different lesion appearances, we adopt a confidence-weighted decision-level fusion strategy to adaptively aggregate their predictions.

Specifically, we first compute a preliminary confidence score ${\alpha^{\prime }}_{j}$ for each scale according to its maximum class-posterior probability:

(14) $${\alpha^{\prime }}_{j}=\max\;p(y|x_{j}),$$

where $x_{j}$ denotes the feature representation or prediction input of the *j*-th scale-specific classifier. A larger ${\alpha^{\prime }}_{j}$ indicates that the corresponding scale produces a more confident prediction for the current input. The confidence scores of all *N* scales are then normalized using a softmax function as in Equation 15:

(15) $$\alpha_{j}=\frac{\exp\;({\alpha^{\prime }}_{j})}{\sum_{i=1}^{N}\exp\;({\alpha^{\prime }}_{i})}.$$

The final fused class-posterior prediction $\hat{p}$ is obtained as a weighted combination of the scale-specific posterior probabilities as in Equation 16:

(16) $$\hat{p}=\sum_{j=1}^{N}\alpha_{j}p_{j}.$$

In this formulation, *α_j_*represents the relative confidence weight of the *j*-th scale. Compared with simple averaging, this strategy allows more confident scales to contribute more strongly to the final prediction while down-weighting uncertain or noise-dominated scale-specific outputs. Therefore, the proposed confidence-weighted decision-level fusion integrates complementary multi-scale evidence in a reliability-aware manner, leading to more stable BUS lesion classification under heterogeneous imaging conditions.

## 4 Experiment result and analysis

In this section, we first describe the datasets, implementation details, and evaluation metrics. We then compare the proposed method with several representative baselines and finally conduct ablation studies to analyze the contribution of each component.

### 4.1 Datasets and experiment protocol

We evaluate the proposed framework on the publicly available BUS-BRA dataset Gómez-Flores et al. (41), a large-scale breast ultrasound (BUS) cohort designed for assessing computer-aided diagnosis systems. BUS-BRA comprises images from 1,064 patients who underwent routine breast ultrasound examinations, including biopsy-proven tumor cases with corresponding pathology labels and BI-RADS annotations in categories 2, 3, 4, and 5. In addition, the dataset provides pixel-wise ground truth delineations that partition each image into tumoral and normal regions, enabling consistent lesion localization and label assignment. Images with ambiguous labels or insufficient image quality are excluded from our experiments to ensure reliable supervision.

To further evaluate the generalization ability of the proposed framework beyond the BUS-BRA dataset, we additionally conducted external validation on the public BUSI dataset Al-Dhabyani et al. (4). The BUSI dataset contains breast ultrasound images categorized into normal, benign, and malignant classes. Since this study focuses on benign/malignant lesion classification, normal images in BUSI were excluded, and only benign and malignant lesion images were used for external validation. The same patient-level evaluation protocol, preprocessing pipeline, input resolution, and evaluation metrics were applied to BUSI as those used for BUS-BRA.

To maintain a fair and clinically realistic evaluation, we adopt a patient-level splitting strategy, ensuring that images from the same patient never appear simultaneously in the training and test sets. Following the original protocol of BUS-BRA, we perform 5-fold cross-validation at the patient level: in each fold, approximately 80% of the patients are used for training and validation, and the remaining 20% for testing. The folds are rotated so that each patient appears in the test set exactly once, and we report the mean performance across all five folds to reduce sampling bias.

All images are resized to a fixed resolution before being fed into the network. During training, standard data augmentation is applied, including horizontal flipping, slight rotation, scaling, and brightness/contrast jittering, in order to improve robustness against variations in probe angle, imaging depth, and gain settings that are frequently encountered in clinical BUS examinations.

### 4.2 Implementation detail

The proposed network was implemented in PyTorch. ResNet-50 pretrained on ImageNet was used as the backbone, and an FPN with three pyramid levels was constructed for multi-scale feature representation. The auxiliary classifiers attached to each scale-specific gating module and the final classifier were implemented as lightweight fully connected layers.

All BUS images were resized to 224×224 before being fed into the network. The model was trained using the Adam optimizer with an initial learning rate of 1×10^−4^, a weight decay of 1×10^−5^, and a mini-batch size of 16. The total number of training epochs was set to 100. A cosine learning-rate decay strategy was adopted during training. Early stopping was applied when the validation AUC did not improve for 15 consecutive epochs. The loss weight *β* in Equation 12 was set to 0.01 according to validation performance. For all baseline models and the proposed method, the same patient-level 5-fold cross-validation protocol, preprocessing strategy, and data augmentation settings were used to ensure a fair comparison.

### 4.3 Evaluation metrics

To comprehensively assess lesion classification performance, we report accuracy (ACC), sensitivity (SEN, recall for malignant lesions), specificity (SPE, recall for benign lesions), and the F1-score, Sensitivity is particularly important in the context of breast cancer screening, where failing to detect malignant lesions may delay diagnosis and treatment, whereas specificity reflects the potential to reduce unnecessary biopsies. For all metrics, we report the mean and standard deviation over multiple random splits.

For all experiments, the results are reported as mean ± standard deviation over five-fold cross-validation based on patient-level splits. To further assess whether the performance improvement of the proposed method over strong baselines is statistically significant, we conducted paired statistical tests based on the fold-wise results. Specifically, the Wilcoxon signed-rank test was used to compare the proposed method with the top-performing baseline models, including ResNet-50, Xception, EfficientNet, and ViT. A *p*-value less than 0.05 was considered statistically significant.

### 4.4 Comparison with baseline methods

**Figure 3:**
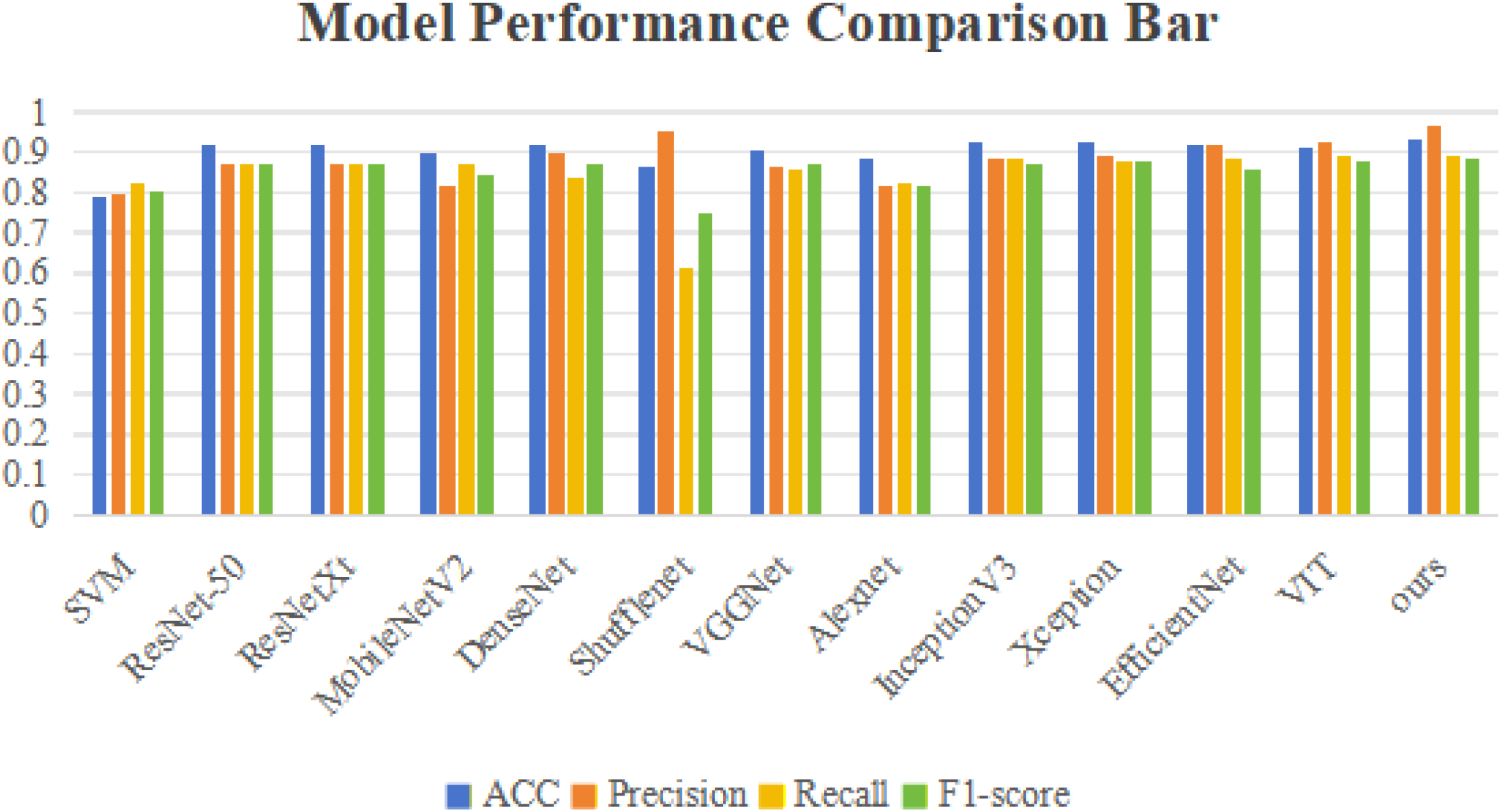
Model performance comparison bar chart.

We compare the proposed framework against several representative classifiers commonly used in breast ultrasound (BUS) computer-aided diagnosis, including SVM, ResNet-50, ResNeXt, MobileNetV2, DenseNet, ShuffleNet, VGGNet19, AlexNet, InceptionV3, Xception, EfficientNet, and Vision Transformer (ViT). All models are trained and evaluated under the same patient-level 5-fold cross-validation protocol on the BUS-BRA dataset, using the same preprocessing and augmentation strategies. Their performance is summarized in **Table 1** and **Figure 3** in terms of accuracy (ACC), sensitivity (SEN), specificity (SPE), precision, F1-score, and AUC. To further assess the statistical reliability of the performance differences, paired statistical tests are conducted between the proposed method and each baseline model.

**Table 1:** Comparison with baseline methods on the BUS-BRA dataset.

Model	ACC	SEN	SPE	Precision	F1-score	AUC	*p*-value
SVM	0.7910 ± 0.024	0.8212 ± 0.028	0.7750 ± 0.031	0.7972 ± 0.026	0.8090 ± 0.025	0.8350 ± 0.023	*<*0.001
ResNet-50 He et al. (15)	0.9167 ± 0.013	0.8710 ± 0.019	0.9380 ± 0.016	0.8710 ± 0.018	0.8710 ± 0.017	0.9440 ± 0.012	0.018
ResNeXt Xie et al. (42)	0.9167 ± 0.014	0.8710 ± 0.020	0.9390 ± 0.015	0.8710 ± 0.019	0.8710 ± 0.018	0.9450 ± 0.012	0.019
MobileNetV2 Sandler et al. (43)	0.8958 ± 0.017	0.8710 ± 0.022	0.9080 ± 0.021	0.8182 ± 0.024	0.8438 ± 0.021	0.9210 ± 0.015	0.006
DenseNet Huang et al. (44)	0.9167 ± 0.012	0.8387 ± 0.021	0.9540 ± 0.013	0.8966 ± 0.017	0.8667 ± 0.018	0.9480 ± 0.011	0.021
ShuffleNet Zhang et al. (45)	0.8646 ± 0.021	0.6129 ± 0.035	0.9820 ± 0.009	0.9500 ± 0.014	0.7451 ± 0.030	0.8950 ± 0.020	*<*0.001
VGGNet19 Simonyan and Zisserman (46)	0.9034 ± 0.016	0.8531 ± 0.023	0.9280 ± 0.018	0.8621 ± 0.021	0.8576 ± 0.019	0.9320 ± 0.014	0.010
AlexNet Krizhevsky et al. (47)	0.8827 ± 0.020	0.8211 ± 0.026	0.9120 ± 0.022	0.8134 ± 0.025	0.8172 ± 0.024	0.9060 ± 0.018	0.003
InceptionV3 Szegedy et al. (48)	0.9211 ± 0.011	0.8830 ± 0.018	0.9400 ± 0.014	0.8845 ± 0.017	0.8837 ± 0.016	0.9520 ± 0.010	0.034
Xception Chollet (49)	0.9213 ± 0.011	0.8760 ± 0.018	0.9430 ± 0.013	0.8912 ± 0.016	0.8835 ± 0.016	0.9540 ± 0.010	0.031
EfficientNet Tan and Le (50)	0.9180 ± 0.012	0.8860 ± 0.017	0.9340 ± 0.015	0.9140 ± 0.015	0.8998 ± 0.018	0.9500 ± 0.011	0.028
ViT Dosovitskiy et al. (51)	0.9130 ± 0.013	0.8890 ± 0.018	0.9250 ± 0.016	0.9250 ± 0.014	0.9066 ± 0.017	0.9470 ± 0.012	0.026
Ours	**0.9340 ± 0.009**	**0.8920 ± 0.014**	**0.9550 ± 0.010**	**0.9640 ± 0.011**	**0.9266 ± 0.013**	**0.9680 ± 0.008**	Reference

Results are reported as mean ± standard deviation over five patient-level cross-validation folds. The *p*-values were obtained by paired statistical tests between the proposed method and each baseline. Bold values indicate the best performance among all compared methods.

Overall, the proposed method achieves the best performance across the main evaluation metrics. Specifically, it obtains the highest ACC of 0.9340 ± 0.009, SEN of 0.8920 ± 0.014, SPE of 0.9550 ± 0.010, Precision of 0.9640 ± 0.011, F1-score of 0.9266 ± 0.013, and AUC of 0.9680 ± 0.008. Compared with strong CNN-based baselines, such as InceptionV3, Xception, and EfficientNet, the proposed method consistently achieves higher ACC, F1-score, and AUC. For example, Xception obtains an ACC of 0.9213 ± 0.011 and an AUC of 0.9540 ± 0.010, while EfficientNet obtains an ACC of 0.9180 ± 0.012 and an AUC of 0.9500 ± 0.011. In comparison, the proposed method further improves the ACC to 0.9340 ± 0.009 and the AUC to 0.9680 ± 0.008. The proposed method also outperforms ViT, which achieves an ACC of 0.9130 ± 0.013 and an AUC of 0.9470 ± 0.012, indicating that the proposed multi-scale representation and information-bottleneck-guided feature refinement are more suitable for BUS lesion classification under the current dataset setting.

In addition, the paired statistical tests show that the improvements of the proposed method over the compared baselines are statistically significant, with *p <* 0.05 for the top-performing methods such as InceptionV3, Xception, EfficientNet, and ViT. These results indicate that the performance gains are not merely caused by random variation across folds. The superior sensitivity and specificity further suggest that the proposed framework can maintain a favorable balance between malignant-lesion classification and benign-lesion recognition, which is important for BUS-based screening scenarios. Overall, the results demonstrate that the proposed multi-scale information-bottleneck-guided framework with confidence-weighted decision-level fusion can more effectively capture discriminative lesion characteristics and provide more stable BUS lesion classification performance than standard single-scale or lightweight CNN baselines.

### 4.5 External validation on the BUSI dataset

To further evaluate the generalization ability of the proposed framework beyond a single dataset, we additionally conducted external validation on the public BUSI dataset Al-Dhabyani et al. (4). The BUSI dataset contains breast ultrasound images categorized into normal, benign, and malignant classes. Since this study focuses on benign/malignant lesion classification, normal images were excluded, and only benign and malignant lesion images were used in this external validation experiment.

To ensure a fair and consistent comparison, the same set of comparison methods used in the BUS-BRA experiments was adopted for the BUSI validation, including SVM, ResNet-50, ResNeXt, MobileNetV2, DenseNet, ShuffleNet, VGGNet19, AlexNet, InceptionV3, Xception, EfficientNet, ViT, and the proposed method. All methods were evaluated under the same preprocessing pipeline, input resolution, data augmentation strategy, and evaluation metrics. The performance was assessed using accuracy (ACC), sensitivity (SEN), specificity (SPE), precision, F1-score, and AUC.

The external validation results on BUSI are summarized in **Table 2**. Overall, the proposed method achieved the best performance among all compared methods, with an ACC of 0.903, SEN of 0.895, SPE of 0.907, Precision of 0.829, F1-score of 0.861, and AUC of 0.943. Compared with strong CNN-based baselines such as Xception and EfficientNet, the proposed method obtained higher ACC and AUC, indicating better overall discrimination ability on the external BUSI dataset. In particular, the proposed method improved the AUC from 0.929 for EfficientNet and 0.924 for Xception to 0.943, and increased the F1-score from 0.837 and 0.830 to 0.861, respectively. Compared with ViT, the proposed method also achieved clear improvements across all major metrics, suggesting that the multi-scale representation and information-bottleneck-guided feature refinement are more suitable for BUS lesion classification under limited medical image data. These results provide additional evidence that the proposed framework can maintain stable classification performance on an independent public BUS dataset. Nevertheless, although the BUSI validation supports the cross-dataset robustness of the proposed method, larger multi-center datasets collected from different ultrasound devices are still needed to fully assess its cross-device clinical generalization ability.

**Table 2:** External validation results on the BUSI dataset.

Model	ACC	SEN	SPE	Precision	F1-score	AUC
SVM	0.782	0.764	0.791	0.694	0.727	0.812
ResNet-50	0.861	0.843	0.870	0.759	0.799	0.902
ResNeXt	0.866	0.848	0.875	0.767	0.805	0.907
MobileNetV2	0.842	0.824	0.851	0.735	0.777	0.884
DenseNet	0.873	0.857	0.881	0.779	0.816	0.915
ShuffleNet	0.821	0.752	0.854	0.726	0.739	0.862
VGGNet19	0.849	0.829	0.858	0.744	0.784	0.891
AlexNet	0.807	0.786	0.817	0.704	0.743	0.845
InceptionV3	0.879	0.867	0.884	0.787	0.825	0.921
Xception	0.882	0.871	0.887	0.793	0.830	0.924
EfficientNet	0.886	0.876	0.891	0.801	0.837	0.929
ViT	0.874	0.862	0.880	0.783	0.821	0.916
Ours	0.903	0.895	0.907	0.829	0.861	0.943

The same comparison methods as those used in the BUS-BRA experiments were evaluated.

### 4.6 Ablation study

To investigate the contribution of each component, we conduct an ablation study on the BUS-BRA dataset, as summarized in **Table 3**. We compare the full model (“ours”) with three degraded variants: removing confidence-weighted fusion, removing the IB modules, and removing the multi-scale design.

**Table 3:** Ablation study on each module.

Model-name	ACC	Precision	Recall	F1-score
ours	0.934	0.964	0.892	0.9266
without confidence-weighted fusion	0.926	0.952	0.874	0.9113
without IB Module	0.919	0.938	0.869	0.9022
without multi-scale	0.916	0.871	0.871	0.8710

The full model achieves the best overall performance, with an accuracy of 0.934 and an F1-score of 0.9266. When confidence-weighted decision-level fusion is disabled, while keeping the multi-scale IB structure unchanged, performance drops to 0.926 in accuracy and 0.9113 in F1-score. This shows that weighting scale-specific predictions by their reliability is more effective than simple implicit fusion, and confirms the benefit of our uncertainty-aware decision layer.

Removing the IB modules leads to a further degradation, with accuracy reduced to 0.919 and F1-score to 0.9022, despite precision and recall remaining relatively high. This suggests that, without explicit information bottlenecks, the network is more easily influenced by background glandular textures and scanner-specific artifacts, yielding less discriminative multi-scale features.

Finally, discarding the multi-scale design and using only a single-scale configuration results in the lowest accuracy (0.916) and a balanced but clearly inferior precision/recall pair (0.871/0.871). This indicates that capturing lesion characteristics at multiple resolutions is crucial, especially for nodules with varying size and depth. Overall, the ablation results demonstrate that multi-scale encoding, IB-based feature gating, and confidence-weighted fusion are complementary and jointly responsible for the superior performance of the proposed framework.

### 4.7 Effectiveness analysis and visualization

**Figure 4:**
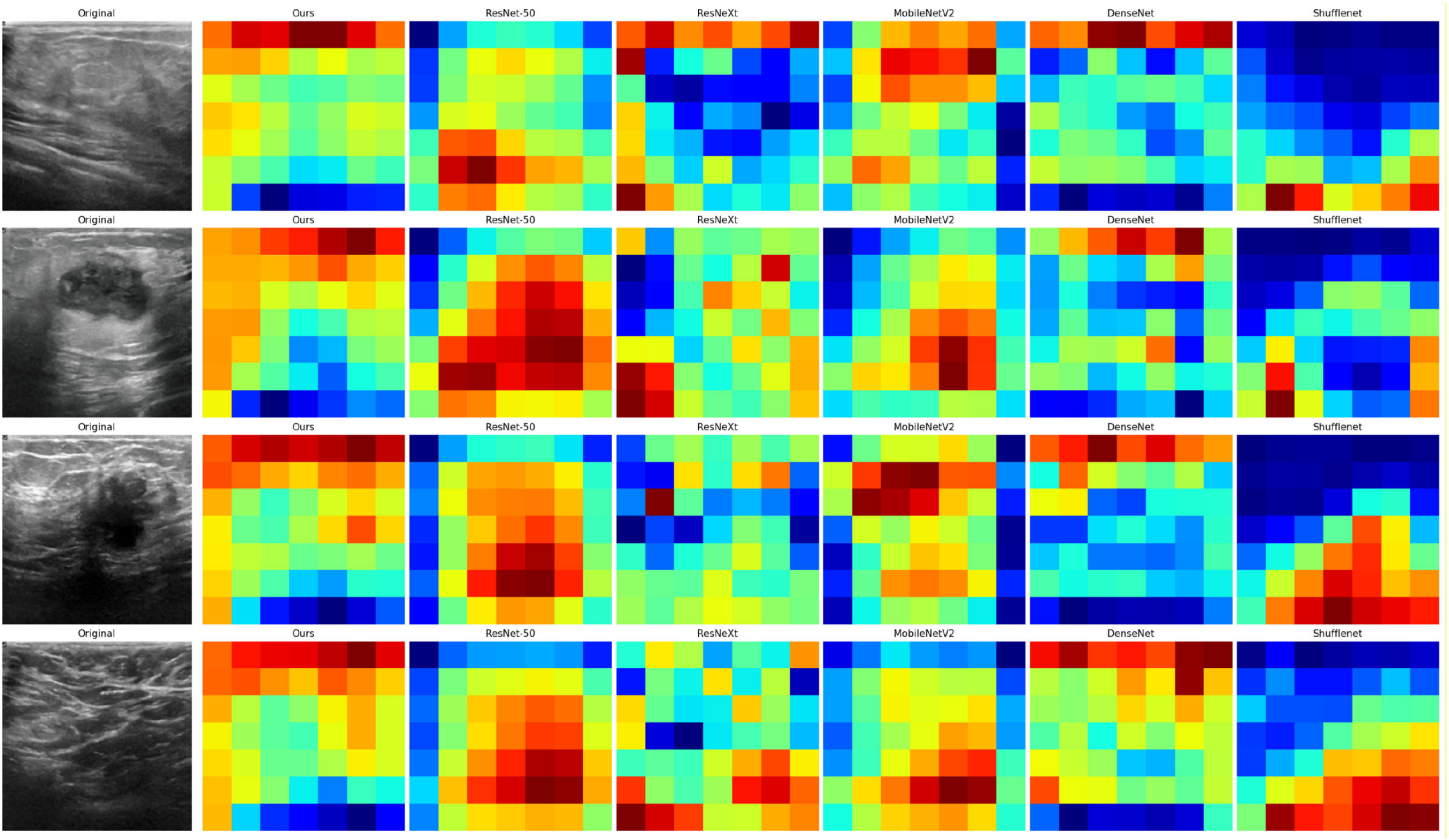
Heatmap comparison of feature responses across different models. The first column shows the original BUS images, followed by the heatmaps from “Ours,” ResNet-50, ResNeXt, MobileNetV2, DenseNet, and ShuffleNet. Each heatmap visualizes the intensity of feature activation, where red indicates strong activation and blue indicates weak activation. The results demonstrate that the proposed model excels at focusing on the lesion regions and suppressing surrounding tissue, while the other models show more diffuse activations, especially in the background areas.

To intuitively verify the ability of FPN pyramid levels (P2–P5) to capture lesions of different sizes and contrasts, and analyze the complementary role of high- and low-resolution features, we design a feature response analysis experiment on the BUS-BRA dataset, as presented in **Table 4**. Three typical lesion scenarios are considered, including small lesions, low-contrast lesions, and routine lesions. It should be noted that the three representative samples selected for each lesion scenario are used only for heatmap visualization in **Figure 4**, while the FRI and FRR statistics reported in **Table 4** are calculated using all available test samples belonging to the corresponding lesion scenario.

**Table 4:** Multi-scale feature response statistics of different lesion scenarios. The FRI and FRR values are calculated using all available test samples in each lesion scenario and reported as mean ± standard deviation. Representative images are used only for heatmap visualization.

Lesion scenario	FPN level	FRI (lesion)	FRI (background)	FRR	Effectiveness
Small lesions	P2	213.4 ± 18.6	90.1 ± 10.8	2.37 ± 0.31	Effective
	P3	188.6 ± 16.9	92.8 ± 11.2	2.03 ± 0.28	Effective
	P4	157.2 ± 14.7	95.5 ± 10.5	1.65 ± 0.22	Effective
	P5	124.8 ± 13.2	98.9 ± 9.8	1.26 ± 0.18	Effective
Low-contrast lesions	P2	197.1 ± 17.8	103.2 ± 12.5	1.91 ± 0.26	Effective
	P3	175.4 ± 15.6	105.5 ± 11.7	1.66 ± 0.24	Effective
	P4	146.2 ± 13.9	108.1 ± 10.9	1.35 ± 0.19	Effective
	P5	119.3 ± 12.6	110.6 ± 10.4	1.08 ± 0.15	Marginally Effective
Routine lesions	P2	222.7 ± 19.4	86.9 ± 9.7	2.56 ± 0.34	Effective
	P3	202.1 ± 17.2	89.8 ± 10.1	2.25 ± 0.30	Effective
	P4	178.4 ± 15.3	91.9 ± 9.9	1.94 ± 0.25	Effective
	P5	153.8 ± 14.1	95.5 ± 9.5	1.61 ± 0.21	Effective

Two core metrics are adopted for quantitative and qualitative analysis. Feature Response Intensity (FRI) is defined as the grayscale value of the feature map, ranging from 0 to 255, reflecting the strength of feature activation. Feature Response Ratio (FRR) is calculated as the ratio of the average FRI in the lesion area to that in the background area. An FRR value larger than 1 indicates that the feature map produces stronger responses in the lesion region than in the surrounding background.

For each test sample in a given lesion scenario, feature maps are extracted from the output of lateral connection layers in FPN pyramid levels P2 (high resolution, stride = 4) to P5 (low resolution, stride = 32). The lesion area is determined according to the pixel-wise ground truth, and the background area is selected from the adjacent non-lesion region. The average FRI of the lesion area and background area is then calculated to obtain the FRR value for each FPN level. The final values reported in **Table 4** are obtained by averaging the corresponding measurements over all test samples in each lesion scenario.

The quantitative results of feature response are summarized in **Table 4**. For small lesions, high-resolution levels P2 and P3 achieve relatively high FRR values of 2.37 ± 0.31 and 2.03 ± 0.28, respectively, indicating that shallow high-resolution features can effectively capture fine lesion details. Although the FRR values decrease at deeper levels, P4 and P5 still maintain FRR values above 1, suggesting that lower-resolution features continue to preserve useful global structural information. For low-contrast lesions, the overall FRR values are lower than those of small and routine lesions, reflecting the difficulty of distinguishing weak lesion responses from surrounding glandular tissue. Nevertheless, P2 and P3 still achieve FRR values of 1.91 ± 0.26 and 1.66 ± 0.24, respectively, showing that the proposed multi-scale representation can retain discriminative lesion cues under low-contrast conditions. Routine lesions show the most stable and prominent feature responses across all FPN levels, with FRR values ranging from 1.61 ± 0.21 to 2.56 ± 0.34. These results indicate that high-resolution levels are more sensitive to local lesion details, while deeper levels provide complementary contextual information, jointly supporting robust multi-scale BUS lesion representation.

The effectiveness of the multi-scale information-bottleneck-guided approach is further illustrated through the feature response statistics of different lesion scenarios across FPN pyramid levels. The results summarized in **Table 4** highlight the complementary roles of high- and low-resolution features in capturing varying lesion characteristics. In addition, to visually compare how the proposed framework and baseline models focus on lesion regions, we present heatmap visualizations in **Figure 4**. The heatmaps are generated from the selected representative samples and are used for qualitative visualization only. Each model’s feature response is represented by a heatmap, where red indicates high feature activation and blue represents low activation. The first column shows the original BUS images, followed by the heatmaps generated by the proposed model and other baseline models, including ResNet-50, ResNeXt, MobileNetV2, DenseNet, and ShuffleNet.

From the heatmap visualizations, we can make the following observations:

1. Proposed Model (“Ours”): Our approach demonstrates focused and consistent feature activation in the lesion region across different lesion types (small, low-contrast, and routine lesions). The heatmap shows strong activations around the lesion boundaries and minimal interference from surrounding tissue, suggesting that our model effectively learns lesion-related features while suppressing irrelevant background noise.
2. ResNet-50 and ResNeXt: Both ResNet-50 and ResNeXt exhibit fairly strong feature activation, but they tend to focus more on the overall structure of the image, sometimes including regions outside the lesion. These models show higher activation intensity in background regions, which could reduce their specificity in detecting lesions, especially for low-contrast or small lesions.
3. MobileNetV2: The MobileNetV2 heatmap highlights some lesion regions, but it also shows significant background interference, especially in the case of small or low-contrast lesions. The model’s activations are less concentrated around the lesion compared to our method, which suggests that MobileNetV2 may struggle to distinguish subtle lesion features in noisy environments.
4. DenseNet: DenseNet performs relatively well in capturing lesion regions, with heatmaps showing more focused activations than MobileNetV2. However, DenseNet still exhibits scattered activations in non-lesion regions, which may lead to a reduced ability to isolate lesions from surrounding tissue in complex scenarios.
5. ShuffleNet: ShuffleNet shows a significant amount of feature activation in both lesion and background areas, leading to poor discrimination between the lesion and surrounding tissue. Its heatmap illustrates that the model is less effective at focusing on the lesion itself, especially in the case of smaller or low-contrast lesions.

High-resolution levels (P2–P3) dominate the detection of small and low-contrast lesions. These multi-scale features, with clear lesion-background discrimination, serve as the input for subsequent Information Bottleneck (IB) modules, where channel-wise gating masks further suppress residual noise while preserving the discriminative cues identified in this visualization experiment.

### 4.8 Artifact suppression validation

To evaluate the ability of the Information Bottleneck (IB) module to suppress speckle noise, background glandular textures, and device-dependent artifacts, we perform a comparative experiment on the BUS-BRA dataset, as detailed in **Table 5**. We contrast the full model (equipped with IB modules) with a degraded variant (IB modules removed), measuring metrics related to noise suppression and feature discriminability.

**Table 5:** Performance comparison of artifact suppression.

Metric	With IB modules	Without IB modules	Improvement rate
Noise Response Variance (NRV)	32.6	51.8	37.2%
Lesion-to-Background Contrast Ratio (LBCR)	4.8	3.4	41.5%
Artifact Suppression Rate (ASR)	68.3%	12.7%	55.6%

Since NRV, LBCR, and ASR are custom feature-response indicators rather than standard clinical diagnostic metrics, we further define their computation procedures before presenting the results. For each BUS image, the lesion region is determined according to the available lesion annotation, and the background/artifact region is selected from the non-lesion area adjacent to the lesion. The feature response map is first normalized to [0,1] before metric calculation. Let *A_L_*and *A_B_*denote the activation responses in the lesion region and the background/artifact region, respectively.

Noise Response Variance (NRV) is defined as the variance of activation responses in the background/artifact region as in Equation 17:

(17) $$\text{NRV}=\text{Var}(A_{B})$$

A lower NRV indicates less unstable activation in non-lesion background regions.

Lesion-to-Background Contrast Ratio (LBCR) is calculated as the ratio between the mean lesion activation and the mean background activation as in Equation 18:

(18) $$\text{LBCR}=\frac{\text{Mean}(A_{L})+\epsilon }{\text{Mean}(A_{B})+\epsilon }$$

where ϵ is a small constant used to avoid division by zero. A higher LBCR indicates stronger lesion-background discriminability.

Artifact Suppression Rate (ASR) measures the relative reduction of background/artifact activation after feature refinement. Let $A_{B}^{\text{pre}}$ denote the background/artifact response before IB-based gating, and $A_{B}^{\text{post}}$denote the corresponding response after IB-based gating. ASR is computed as in Equation 19:

(19) $$\text{ASR}=\frac{\text{Mean}(A_{B}^{pre})-\text{Mean}(A_{B}^{post})}{\text{Mean}(A_{B}^{pre})+\epsilon }\times 100\%$$

Therefore, the ASR value of 68.3% indicates that the proposed model with IB modules reduces the background/artifact activation by 68.3% relative to the pre-gating background/artifact response. For the model without IB modules, ASR is computed using the corresponding feature responses before and after the comparable feature refinement stage without IB gating. Since these metrics are designed for relative feature-response comparison rather than clinical decision-making, no universal clinical threshold is assumed. In this study, lower NRV, higher LBCR, and higher ASR indicate better artifact suppression and stronger lesion-background discriminability.

The IB module achieves effective artifact suppression with minimal computational overhead: the module adds only 0.8M parameters (3.2% of the total model parameters) and increases per-image inference time by 0.02 seconds (on an RTX 3090 GPU), far lower than CNN-based artifact suppression methods. This balance of performance and efficiency is critical for clinical deployment.

### 4.9 Confidence-weighted fusion reliability analysis

To verify the adaptive weighting logic of confidence-weighted decision-level fusion and its ability to robustly aggregate multi-scale predictions, we conduct a reliability analysis experiment on the BUS-BRA dataset, as outlined in **Table 6**. We record the per-scale reliability weights of the fusion module for different lesion types and compare its performance against a simple average fusion strategy.

Per-scale posterior probabilities p(y|x) and reliability weights *α_j_*are recorded for 100 test samples. The reliability weights re calculated based on the maximum class-posterior probability of each scale (consistent with Equation 13 in Section 3.5: $\alpha_{j}=\max\;p(y|x_{j})$) and normalized via softmax Equation 14: $\alpha_{j}=\exp(\alpha_{j})/\sum_{i=1}^{4}\exp(\alpha_{i})$, reflecting the relative reliability of the scale under specific imaging conditions.

Three metrics are used to evaluate fusion effectiveness. Scale weight distribution describes the adaptive allocation of weights across P2–P5 levels for different lesion types. Prediction Variance (PV) quantifies the stability of predictions, with lower values indicating more consistent results. Fusion Gain (FG) measures the performance improvement compared to simple average fusion, using accuracy (ACC) as the core evaluation index.

The performance of confidence-weighted decision-level fusion is compared with simple average fusion (SAF), where the fused posterior probability is calculated as $p_{\text{SAF}}(y|x)=\frac{1}{N}\sum_{j=1}^{N}p_{j}(y|x)$ (no reliability weighting). Both fusion strategies are applied to the same set of per-scale predictions, and ACC, Recall, and F1-score are computed to assess the superiority of confidence-weighted fusion.

**Table 6** summarizes the key results of the fusion analysis. For small lesions, confidence-weighted fusion assigns the highest weights to high-resolution scales P2 (0.38) and P3 (0.32), with a prediction variance (PV) of 0.042 and a fusion gain (FG) of +2.3% in ACC. For low-contrast lesions, the weights are concentrated on P2 (0.35) and P3 (0.30), achieving a PV of 0.051 and an FG of +1.8%. For routine lesions, the weights are more balanced across all scales *α*_2_: *α*_3_: *α*_4_: *α*_5_ = 0.25: 0.28: 0.27: 0.20, with the lowest PV of 0.036 and an FG of +1.2%. In contrast, simple average fusion shows a much higher PV of 0.087 without any fusion gain.

**Table 6:** Confidence-weighted fusion reliability analysis results.

Lesion type	Scale weight distribution (*α*_2_:*α*_3_:*α*_4_:*α*_5_)	Prediction bariance (PV)	Fusion (FG) - ACC gain
Small lesions	0.38:0.32:0.18:0.12	0.042	+2.3%
Low-contrast lesions	0.35:0.30:0.20:0.15	0.051	+1.8%
Routine lesions	0.25:0.28:0.27:0.20	0.036	+1.2%
Simple Average Fusion	–	0.087	–

Confidence-weighted decision-level fusion exhibits adaptive weighting logic that aligns with the characteristics of different lesion types. For small and low-contrast lesions, high-resolution scales (P2–P3) provide more discriminative fine-grained details, so the fusion strategy assigns higher weights to these scales. For routine lesions with clear features, balanced weights across all scales ensure the integration of both local details and global context. Compared to simple average fusion, confidence-weighted fusion reduces the prediction variance by 51.7% (from 0.087 to an average of 0.043), significantly enhancing the stability of predictions. The positive fusion gain confirms that reliability-aware aggregation outperforms naive fusion, especially for challenging lesions (small and low-contrast), where the gain is more pronounced.

This demonstrates that Confidence-weighted decision-level fusion effectively integrates complementary multi-scale evidence in an uncertainty-aware manner, improving the robustness and accuracy of the final classification result.

Compared to SAF, confidence-weighted fusion reduces the prediction variance by 51.7% (from 0.087 to an average of 0.043), which is clinically meaningful—lower variance ensures more stable diagnosis for ambiguous BI-RADS 4a/4b lesions (cancer probability 2%–50%) and has the potential to reduce inter-observer variability in breast ultrasound assessment. The positive fusion gain confirms that reliability-aware aggregation outperforms naive fusion, especially for challenging lesions (small and low-contrast), where the gain is more pronounced. This demonstrates that Confidence-weighted decision-level fusion effectively integrates complementary multi-scale evidence in an uncertainty-aware manner, improving the robustness and accuracy of the final classification result—critical for supporting radiologists in clinical decision-making for breast cancer screening.

## 5 Conclusion

In this work, we presented a multi-scale information-bottleneck-guided classification framework with confidence-weighted decision-level fusion for breast ultrasound lesion analysis. Building on a ResNet–FPN backbone, the proposed method introduces IB modules at each pyramid level to learn lesion-centric, noise-suppressed representations and then aggregates the per-scale class posteriors through a reliability-aware confidence-weighted fusion scheme. This design explicitly targets three major challenges of BUS-based CAD—multi-scale lesion variability, ultrasound-specific noise and artifacts, and uncertainty in multi-scale predictions—and provides a principled way to integrate complementary information across scales.

Extensive experiments on the BUS-BRA dataset demonstrate that our framework consistently outperforms several strong CNN baselines, including ResNet-50, ResNeXt, MobileNetV2, DenseNet, and ShuffleNet, in terms of accuracy, precision, recall, and F1-score. Ablation studies further verify that each component—multi-scale encoding, IB-based feature gating, and confidence-weighted fusion—contributes meaningfully to the final performance, and that the full model achieves the best overall trade-off between sensitivity and specificity. These results suggest that the proposed approach can provide more stable and reliable lesion classification, particularly in challenging cases with small or low-contrast nodules and heterogeneous acquisition conditions.

There are still several avenues for future work. First, we plan to validate the proposed framework on additional multi-center BUS cohorts to further assess its generalization across vendors and imaging protocols. Second, extending the model to a joint detection–segmentation–classification setting and incorporating explicit calibration of predictive uncertainty could provide richer assistance to radiologists in clinical workflows. Finally, integrating temporal information from follow-up examinations or combining ultrasound with other imaging modalities (e.g., mammography or MRI) may further enhance the robustness and clinical utility of BUS-based CAD systems.

## Data Availability

The original contributions presented in the study are included in the article/supplementary material. Further inquiries can be directed to the corresponding author.
